# Full-lung simulations of mechanically ventilated lungs incorporating recruitment/derecruitment dynamics

**DOI:** 10.3389/fnetp.2023.1257710

**Published:** 2023-11-02

**Authors:** Haoran Ma, Hideki Fujioka, David Halpern, Jason H. T. Bates, Donald P. Gaver

**Affiliations:** ^1^ Department of Biomedical Engineering, Tulane University, New Orleans, LA, United States; ^2^ Center for Computational Science, Tulane University, New Orleans, LA, United States; ^3^ Department of Mathematics, University of Alabama, Tuscaloosa, AL, United States; ^4^ Larner College of Medicine, University of Vermont, Burlington, VT, United States

**Keywords:** acute respiratory distress syndrome (ARDS), ventilator-induced lung injury (VILI), recruitment and derecruitment (RD), pressure-volume (PV) relationship, multiscale computational model

## Abstract

This study developed and investigated a comprehensive multiscale computational model of a mechanically ventilated ARDS lung to elucidate the underlying mechanisms contributing to the development or prevention of VILI. This model is built upon a healthy lung model that incorporates realistic airway and alveolar geometry, tissue distensibility, and surfactant dynamics. Key features of the ARDS model include recruitment and derecruitment (RD) dynamics, alveolar tissue viscoelasticity, and surfactant deficiency. This model successfully reproduces realistic pressure-volume (PV) behavior, dynamic surface tension, and time-dependent descriptions of RD events as a function of the ventilation scenario. Simulations of Time-Controlled Adaptive Ventilation (TCAV) modes, with short and long durations of exhalation (*T*_*Low*_^*-*^ and *T*_*Low*_^*+*^, respectively), reveal a higher incidence of RD for *T*_*Low*_^*+*^ despite reduced surface tensions due to interfacial compression. This finding aligns with experimental evidence emphasizing the critical role of timing in protective ventilation strategies. Quantitative analysis of energy dissipation indicates that while alveolar recruitment contributes only a small fraction of total energy dissipation, its spatial concentration and brief duration may significantly contribute to VILI progression due to its focal nature and higher intensity. Leveraging the computational framework, the model may be extended to facilitate the development of personalized protective ventilation strategies to enhance patient outcomes. As such, this computational modeling approach offers valuable insights into the complex dynamics of VILI that may guide the optimization of ventilation strategies in ARDS management.

## 1 Introduction

Approximately 200,000 acute respiratory distress syndrome (ARDS) cases occur annually in U.S. (prior to COVID-19), with a mortality of 40% (Rubenfeld et al., 2005; Maybauer et al., 2006). Supportive care centered around mechanical ventilation is the foundation of ARDS treatment, but this itself can damage the lung, leading to ventilator-induced lung injury (VILI). The ARDSNet Protocol reduces tidal volume and plateau pressure to avoid overdistension (OD) of healthy tissue (volutrauma) and applies positive end-expiratory pressure (PEEP) to minimize atelectrauma (Brower et al., 2000). Nevertheless, the mortality rate for ARDS remains unacceptably high.

One of the principal mechanisms driving VILI is the occurrence of repetitive recruitment of lung units that derecruit during expiration. These events generate injurious stresses on the airway and alveolar epithelium that degrade the epithelial barrier in a spatially heterogeneous manner, particularly when pulmonary surfactant function is already compromised by leakage of fluid and proteins from the blood plasma. Once this process begins in earnest it leads to further barrier degradation, increased fluid leakage, and progressively worsening VILI (Jacob et al., 1985; Yamaguchi et al., 2022). Eventually, large areas of the lung become destabilized (Gaver et al., 2020) with concomitant effects on overall lung function. This type of lung injury is known as atelectrauma.

Atelectrauma plays out over a distributed network of airways and alveoli and involves effects manifesting over a wide range of length scales from the molecular (surfactant) to that of the individual epithelial cell to the entire organ. Understanding these complexities and their ultimate consequences for the lung is a considerable challenge. The premise of our research is that meeting these challenges can be greatly facilitated by computational models incorporating the known multi-scale network anatomy and physiology of the lung. Accordingly, in the present study we develop such a model by drawing on our prior modeling work on the interactions between different multiscale pulmonary components (Ma et al., 2020), tethering interactions between the parenchyma and airway wall (Ryans et al., 1985), the non-compliant closure of airways involving liquid plug propagation in 2D airway networks (Ryans et al., 2016), and a time-dependent approach to simulate recruitment and derecruitment (Massa et al., 1985). We demonstrate how the resulting model can provide insight into the nature of VILI development during specific modes of mechanical ventilation, and how this development is linked to energy dissipation within the lung. Our goal is to provide a tool that can assist in identifying personalized ventilation strategies for reducing VILI.

## 2 Materials and methods

Below, we first present our base model in §2.1, which was previously used to simulate a “healthy lung” (Ma et al., 2020). We next modify this base model to create a model of Acute Respiratory Distress Syndrome (ARDS). To accurately simulate the lung’s response to mechanical ventilation in ARDS, we introduce a model of recruitment/derecruitment (RD) behavior at the acinar level through a fractional representation of alveolar collapse within sub-acinar units (§2.2). This behavior dynamically affects the compliance of both specific lung regions and the overall lung. We investigate this behavior by simulating mechanical ventilation using time-controlled adaptive ventilation (TCAV), a form of airway pressure release ventilation (APRV), which has been used to study experimentally the physiological impact of RD and overdistension (OD) in ARDS porcine models (Nieman et al., 2022; Ramcharran et al., 2022). Through our simulations, we examine the occurrence of RD events under different ventilation scenarios and analyze the energy delivery and dissipation that may be associated with VILI. In §5 we provide a Glossary to enhance clarity. In the supplementary, we provide an Appendix that provides specific details of the foundational health lung model based on (Ma et al., 2020).

### 2.1 Base model

**FIGURE 1:**
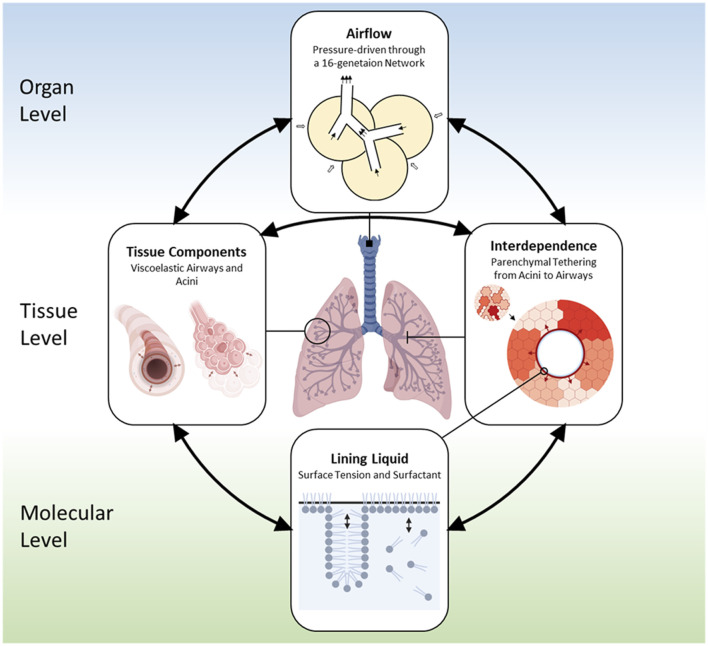
Outline of the base model (Ma et al., 2020). The domain of the lung has a bronchial tree with 16 generations. Components in the model include: 1) Air components, which represent air spaces and airflow; 2) Tissue components, which include compliant airways, acini, and their parenchymal connection; 3) Liquid components, which consist of the liquid lining on the inner tissue wall of the compliant tissue and the soluble surfactant that exist in a multilayer structure.

Our multiscale base model includes spatial scales from the organ-level to the molecular level and allows for temporal resolution to the millisecond timescale. Figure 1 illustrates the interconnections of the different components, including.

1) Airflow in the respiratory tree and acini,
2) Tissue behavior including viscoelasticity,
3) Interdependence between airways and alveoli, and
4) Liquid-lining and surfactant dynamics.

These components create a large set (O (10^6^)) of coupled time-dependent equations that evolve depending on the forcing in the system. For normal breathing, this forcing arises from the time-dependent pleural pressure $P_{PL}\left(t\right)$ , while for mechanical ventilation the airflow and/or pressure at the trachea is prescribed. The rest of the system is updated with an adaptive time increment from the current state. The new state is incrementally solved, and the breathing cycle is tracked. Below, we provide a framework for each of these components and their interactions.

#### 2.1.1 Lung domain

The bronchial tree is created using an anatomically-based space-filling algorithm (Tawhai et al., 2000) that creates an asymmetric network with 16 Horsfield generations as a half-lung within a 3D space. Each airway is compliant, liquid-lined, and terminates with an acinus (described in detail below) that is formed from liquid lined compliant alveoli that are space filling within the lung domain. The Horsfield generation ( n ) for each airway is determined by the algorithm and is used to define the radius ( $R_{AW,\max}$ , the maximum radius) of each airway following (Lambert et al., 1982; Lambert, 1984). In addition, the mechanical properties of airways are described by parameters that are dependent on their generation as explained in the Appendix. Overall, the residual volume (RV) of the half-lung is $V_{RV}=446\text{ mL}$ , and the total lung capacity is approximately 6 $V_{RV}$ .

#### 2.1.2 Airflow in airways and acinus

Airflow in the compliant bronchial tree and alveoli is driven by pressure differences throughout the network. We assume uniform flow in each generation following Eq. 1, where the flowrate $Q_{AW}$ is equal to

$$Q_{AW}=C_{AW}\left(P_{UP}-P_{DN}\right).$$ (1)

Here $C_{AW}$ is the airway conductance, $P_{UP}$ and $P_{DN}$ are the upstream and downstream internal pressure at the ends of an airway, and $Q_{AW}>0$ represents inhalation. $C_{AW}$ follows a Poiseuille relationship with corrections due to disturbed flow in the entrance region (Pedley et al., 1970; van Ertbruggen et al., 1985). $C_{AW}$ is highly dependent on the airway caliber and therefore on the airway compliance. Airway-parenchymal interdependence affects airflow within intrapulmonary airways (see Appendix). A terminal resistance is attached to the terminal airways and is proportional to the acinar volume (0.43 cmH_2_O/(mL/s)/ml), so that the total airway tree resistance is within a realistic range (MacIntyre et al., 2001).

The airway network creates a system of flow equations that satisfy conservation of mass between parent and daughter generations, with the airflow through a terminal airway equal to the airflow into its attached acinus. The endpoints of the network are the trachea and acini, with the acinar pressure linked to its volume through its compliance (see below). As the volume cycles, the internal pressures and airflow vary as a function of both the forcing and the compliance of the system, the latter being determined by tissue components and interdependence, which are described next.

#### 2.1.3 Compliant tissue components and interdependence

Tissue components are comprised of airways and alveoli. The bronchial tree consists of airways that are modeled as compliant vessels with a morphology based on (Lambert, 1984). The airway compliance is described by a tube law that is a function of the local airway transmural pressure, $P_{TM,AW}.$ Similarly, following (Fujioka et al., 2013), the static PV relationship of the acinus is expressed as $V_{AC,norm}=\frac{V_{AC}}{V_{RV,AC}}=g_{PV}\left(P_{TM,AC}\right)$ , where $P_{TM,AC}$ is the acinar transmural pressure. The PV relationship is modified to include alveolar viscoelasticity through inclusion of a three-element Maxwell model (see 6.2).

The mechanical interdependence between airways and alveoli is a critical feature that can help to stabilize the lung (Anafi and Wilson, 2001), though it may also result in localized stress concentrations in the parenchyma (Vlahakis and Hubmayr, 2005). We model interdependence based upon our earlier finite-element analysis (Ryans et al., 1985), which established that the airway transmural pressure $P_{TM,AW}$ can be defined by the relationship

$$P_{TM,AW}=P_{AW}\;–2G_{eff}\left(\frac{∆R}{R}\right)-P_{PL}-P_{\gamma ,AW}.$$ (2)

Here $P_{AW}$ is the airway internal pressure, $P_{PL}$ is the pleural pressure, and $P_{\gamma ,AW}$ is the Laplace pressure drop due to the dynamic surface tension ( γ ) of the airway lining liquid.

Interdependence arises from the term $2G_{eff}\left(\frac{∆R}{R}\right)$ , which describes the compliance effect from surrounding parenchyma where $\frac{∆R}{R}$ is the fractional airway radius change from the uniform strain state. $G_{eff}$ is the effective shear modulus of the parenchymal tissue that surrounds the airway. $G_{eff}$ is a function of $\bar{P_{TP}}$ , which is the average transpulmonary pressure in the surrounding acini within the defined region of interest (Ryans et al., 1985; Ma et al., 2020). This plays a role in the ARDS model because there will be regional variation of $P_{TP}$ resulting from RD behavior.

For acini, the transmural pressure $P_{TM,AC}$ is

$$P_{TM,AC}=P_{AC}-P_{PL}-P_{\gamma ,AC},$$ (3)

where $P_{AC}$ is the acinar internal air pressure, and $P_{\gamma ,AC}$ is the Laplace pressure drop due to the dynamic surface tension ( γ ) of the alveolar lining liquid.

#### 2.1.4 Lining fluid and surfactant components

Because alveoli are small, the dynamic surface tension of the lining fluid plays a very large role in lung mechanics. We model soluble surfactant as existing in a bulk and a multilayer structure at the air-liquid interface where transport between the bulk and interface dynamically modifies the surface tension, as has been described in (Krueger and Gaver, 2000) and in our healthy lung model (Ma et al., 2020) (see Supplementary Material).

As stated above, surface tension affects the compliant response of the tissue components. And, in turn, the change in tissue geometry affects the air-liquid surface area which dynamically modulates the surfactant distribution which influences the mechanical stresses. This physicochemical feedback mechanism contributes to the pressure-volume behavior observed experimentally (Mead et al., 1957) and is replicated in our healthy lung model (Ma et al., 2020).

### 2.2 The ARDS model—An acinar recruitment/derecruitment model

Given the baseline model described above, we now modify this with pathological characteristics as a model of ARDS. In this model, we simulate two aspects of ARDS: 1) surfactant dysfunction, and 2) the resulting RD at the acinar level.

#### 2.2.1 Surfactant dysfunction ( $\phi_{surf}$ )

Surfactant dysfunction is modeled to first order by a reduced surfactant concentration. To do so, we introduce the variable $\phi_{surf}$ that represents the fraction of the normal surfactant mass in the system. When $\phi_{surf}<1,$ this elevates the surface tension, which decreases the compliance and destabilizes alveoli so that RD may occur.

#### 2.2.2 Sub-acinar units

**FIGURE 2:**
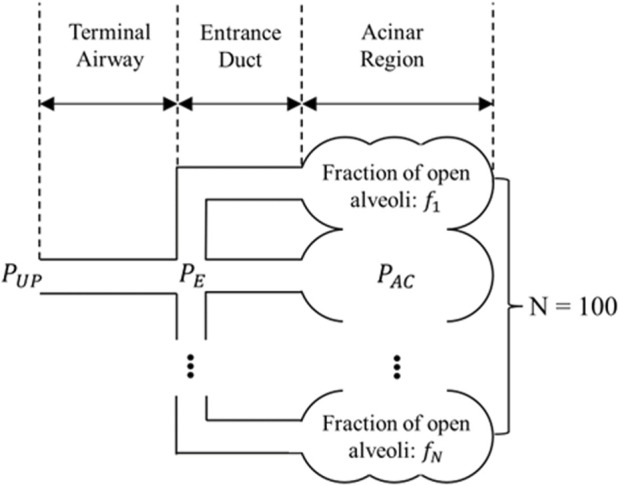
Sub-acinar units (sACs) connected to a terminal airway. Each sAC consists of an entrance duct and its downstream alveolar cluster. These alveolar clusters are interconnected and as a whole follow the PV relationship decribed by Supplementary Eqs 7, 8 in the Appendix.

We subdivide each acinus into N=100 sub-acinar units (sAC), each representing one percent of the alveoli in the acinus. Each sAC is represented by an entrance duct and downstream alveolar components (Figure 2). We assume that the sACs are interconnected so that their ensemble can be described by the viscoelastic model in the Appendix through Supplementary Eqs 7, 8.

Here, $P_{E}$ is the upstream pressure for all entrance ducts, and $P_{AC}$ is the distal pressure in the acinus. Airflow is assumed to be uniform within each duct and satisfies conservation of mass with the terminal airway. That is:

$$Q_{sAC,i}=C_{sAC,i}\left(P_{E}-P_{AC}\right),$$ (4)

$$Q_{AW,t}=\sum Q_{i}=C_{AW,t}\left(P_{UP,t}-P_{E}\right),$$ (5)

where $Q_{sAC,i}$ is the flow into each of the sACs, $C_{sAC,i}$ is the conductance of each entrance duct, $Q_{AW,t}$ is the airflow through the terminal airway, $C_{AW,t}$ is the conductance of the terminal airway, and $P_{UP,t}$ is the upstream air pressure of the terminal airway. By applying the parallel/serial resistor formula, the total air conductance from all entrance ducts, $C_{E}$ , the overall air conductance of the acinus and terminal airway, $C_{AC}$ , and the airflow into the entire acinus, $Q_{AW,t}$ , is, respectively,

$$C_{E}=\sum C_{sAC,i},$$ (6)

$$C_{AC}={\left({C_{AW,t}}^{-1}+{C_{E}}^{-1}\right)}^{-1},$$ (7)

$$Q_{AW,t}=C_{AC}\left(P_{UP,t}-P_{AC}\right).$$ (8)

In our simulations, we assume that all acini are initially open so that the initial $C_{sAc,i}$ are all identical and are calculated from the acinar terminal resistance. We assume $C_{sAC,i}$ changes proportionally to the volume of the recruited acinus during ventilation, as described next.

#### 2.2.3 Acinar recruitment/derecruitment ( $f_{i}$ )

**FIGURE 3:**
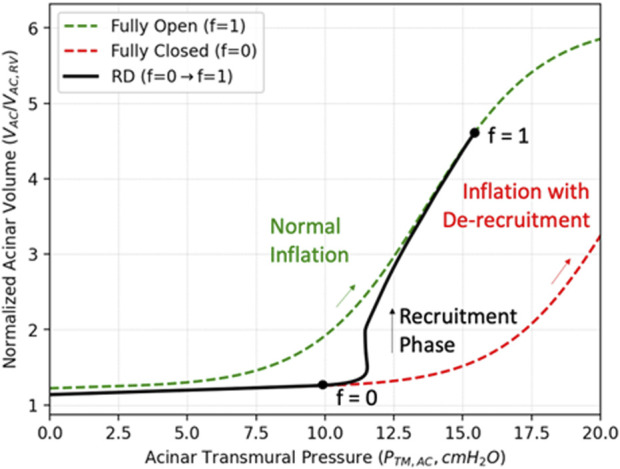
Compliance recovery during recruitment. The green curve describes the PV relationship for a completely recruited acinus during inflation ( f=1) , and the red curve describes the PV relationship for an ARDS derecruited acinus ( f∼0 ), representing a “baby acinus”. With recruitment, *f* increases, and the PV relationship undergoes a dynamic transition towards a healthy acinus (black solid curve).

We now incorporate a model of acinar RD that represents a dynamic conversion from a derecruited to a recruited state, and *vice versa*. To do so, we introduce $f_{i}$ as the fraction of recruited alveoli in sAC_i_. Figure 3 describes two opposite states, 1) the derecruited state ( f∼0 in red) with very low compliance (a “baby acinus,” (Gattinoni and Pesenti, 2005)), and 2) the recruited state ( f=1 in green). These states are modeled by the static PV relationship for different values of recruited volumes (Supplementary Eq. 4). The fractional recruitment process described below, when linked to the viscoelastic model of the acinus (Supplementary Eqs 7, 8), creates the dynamic transition between the fully closed and fully open PV curve, shown in black in Figure 3.

To model this fractional recruitment process, we borrow from the formalism of the Bates “virtual trajectory” model (Massa et al., 1985) to quantify the fraction of recruited alveoli in each sAC with the time-dependent variable $f_{i}$ , that ranges from $f_{i}=0$ (fully closed) to $f_{i}=1$ (fully open).

We assume that the rate of change of the open fraction, f, is proportional to the difference between the transmural pressure at the connection point between sACs and the terminal airway, $P_{TM,E}$ . Two critical pressures exist: 1) $P_{O}$ for opening, and 2) $P_{C}$ for closing, with constants of proportionality $S_{O}$ and $S_{C}$ , respectively. Therefore, $\frac{df}{dt}$ can be expressed as

$$\frac{df}{dt}=\left\{\begin{array}{cc} S_{O}\left(P_{TM,E}-P_{O}\right), & if\;P_{TM,E}>P_{O}\\ S_{C}\left(P_{TM,E}-P_{C}\right), & if\;P_{TM,E}<P_{C}\\ \;0, & otherwise \end{array}\right.,$$ (9)

where

$$P_{TM,E}=P_{TM,AC}+{C_{E}}^{-1}Q_{AW,t}.$$ (10)

The value of f is related to the biophysical properties of the lung during RD. Numerous biofluid mechanic studies have shown that the airway resistance (inverse of conductance) is significantly elevated following derecruitment (Gaver et al., 1990; Halpern and Grotberg, 1994; Fujioka et al., 2016). To model this, we assume the conductance of derecruited entrance airways is

$$C_{sAC,i}=f_{i}C_{sAC,open},$$ (11)

where $C_{sAC,open}$ is the completely recruited sub-acinar region. When $f_{i}=0$ , the sub-acinar region is completely obstructed and is not compliant, so $C_{sAC,i}=0$ . As $f_{i}$ increases during recruitment, $C_{sAC,i}$ increases linearly to $C_{sAC,open}$ when $f_{i}=1$ .

In addition to airway resistance, RD may also affect the acinar compliance (Hickling, 1998). We use an approach comparable to Eq. 11 to describe the compliance (PV relationship) change of the entire interconnected acinar region by

$$V_{AC,norm}=g_{PV}\left[P_{TM,AC}-P_{O}\left(1-\bar{f}\right)\right],$$ (12)

where $g_{PV}$ is the PV relationship described in the Supplementary by Eq. 4, and $\bar{f}$ is the average of $f_{i}$ in all sACs. When all sACs are open ( $\bar{f}=1$ ), the PV of the whole acinus remains unchanged from the healthy lung. When all sACs are derecruited ( $\bar{f}=0$ ), the PV curve shifts to the right by the critical opening pressure $P_{O}$ , so that the acinar compliance decreases. This creates the ‘baby acinus’ relationship shown in red in Figure 3.

#### 2.2.4 Energy dissipation

We estimate the energy dissipation due to RD ( $E_{RD}$ ) as follows. From Eqs.11, 12, RD affects the resistance of the entrance duct as well as the compliance of the alveolar region. Therefore, the energy dissipation due to RD in acinus ( j ) can be quantified by

$$E_{RD,j}=\int \left[{Q_{AW,t,j}}^{2}\left({C_{AC,j}}^{-1}-{C_{AC,open,j}}^{-1}\right)+P_{O}\left(1-{\bar{f}}_{j}\right)Q_{AW,t,j}\right]\;dt,$$ (13)

where $C_{AC,open,j}$ is the overall conductance of the acinus and terminal airway when the they are fully open, and thus ${Q_{AW,t,j}}^{2}\left({C_{AC,j}}^{-1}-{C_{AC,open,j}}^{-1}\right)$ represents the power associated with resistance change due to RD, and the term $P_{O}\left(1-{\bar{f}}_{j}\right)Q_{AW,t,j}$ represents the power associated with the acinar compliance change due to RD.

#### 2.2.5 Parameter estimation

We estimated the closing pressure $P_{C}$ from the stability analysis of compliant fluid-lined tubes associated with the Plateau-Rayleigh instability. We assume that the terminal airways have a radius and compliance equal to the 16th generation airway (Lambert, 1984), with an initial lining fluid thickness of $H=10^{-4}\;cm$ . Given these parameters, we estimate a baseline value $P_{C}=0\;cmH_{2}O$ , since that leads to a critical dimensionless film thickness of $\varepsilon =\frac{H}{R}=0.12$ following the stability analysis of (Halpern and Grotberg, 1994; Fujioka et al., 2016).

We estimate the opening pressure, $P_{O}$ , following the experimental analysis of the reopening of compliant tubes (Gaver et al., 1990), which demonstrated that the critical yield pressure is $P_{yield}∼\frac{8\gamma }{R},$ where γ is the surface tension, and *R* is the radius of the fully inflated tube. We estimate the entrance duct radius R∼0.03 cm , which is approximately the radius of the 16th generation airway (Lambert, 1984). Assuming a surface tension, $\gamma_{eq}=26\;dyn/cm$ , we estimate a baseline $P_{O}=7\;cmH_{2}O$ .

We estimated a baseline $S_{O}=50\;{cmH_{2}O}^{-1}∙s^{-1}$ so that the reopening timescale is approximately 1 *msec*, which is consistent with a 1 kHz crackle frequency (Gavriely, 2019). Following (Massa et al., 1985), we assume that $S_{C}=0.1S_{O}$ , so our baseline $S_{C}=5\;{cmH_{2}O}^{-1}∙s^{-1}.$

In order to prevent all sACs from acting identically, we distributed $P_{O}$ and $P_{C}$ uniformly around their baseline values with a range of ΔP=±1 cmH2O . Likewise, $S_{C}$ and $S_{O}$ were allowed to vary with a multiplier uniformly distributed between 0.5 and 1.5.

### 2.3 Computational tools

The solution vector for an airway object ( i ) is ${AW}_{i}\left(t\right)={\left[P_{TM,AW}\left(t\right)\right]}_{i}$ , where $P_{TM,AW}$ is solved through the airway tube law described by Supplementary Eq. 3.

For an acinus object ( j ), the solution vector is ${AC}_{j}\left(t\right)={\left[V_{AC}\left(t\right),Q_{AC}\left(t\right),P_{M,AC}\left(t\right),f_{1}\left(t\right),f_{2}\left(t\right),\cdots ,f_{N}\left(t\right)\right]}_{j}$ , where $V_{AC}$ and $Q_{AC}$ (the flowrate of the acinus) are solved through mass conservation, $P_{M,AC}$ is solved through the viscoelastic model described in the Appendix, and $f_{1}\left(t\right),f_{2}\left(t\right),\cdots ,f_{N}\left(t\right)$ are solved through Eq. 9.

The lining liquid of each airway and acinus is modeled by a surfactant object. For a surfactant object ( k ), the solution vector is $M_{k}\left(t\right)={\left[M_{p}\left(t\right),M_{s}\left(t\right),M_{b}\left(t\right)\right]}_{k}$ , where $M_{p}$ , $M_{s}$ , $M_{b}$ are the surfactant mass in each partition of the surfactant multilayer structure (Krueger and Gaver, 2000), and solved though Supplementary Eqs 16-18 and Eqs 20-23.

In addition to the individual airway, acinus, and surfactant components, air pressures at each bifurcation in the airway tree are solved by the approach introduced in 2.1. In total, the differential-algebraic equation system includes −150 k unknowns with respective governing equations described above.

High-performance computing simulations were conducted on the Tulane University Cypress supercomputer. The code was written in C++ with the parallel MPI library. The differential-algebraic equation systems solver used in the code was SUNDIALS IDAS solver with adaptive time-stepping (Hindmarsh et al., 2005). The simulation utilized 10 Intel Xeon E5-2,680 v2 CPUs. Wall time for one simulation (60 s of breathing) was up to 6 h.

### 2.4 Simulation protocols

We explore our ARDS model by conducting simulations of time-controlled adaptive ventilation (TCAV), which has been suggested as a personalized method for mechanical ventilation (Nieman et al., 2020). TCAV represents a variant of airway pressure release ventilation (APRV) where the exhalation phase duration ( $T_{Low}$ ) adjusts dynamically, ensuring that exhalation stops at a specific fraction of the maximum expiratory flow rate (MEFR). Given that the expiratory flow characteristics depend on the elasticity of the respiratory system, and since lung compliance decreases in ARDS due to a lack of surfactant, the use of an MEFR criterion to determine $T_{Low}$ might enable the development of a ventilation protocol tailored to individual patients. Other parameters include the airway opening pressure ( $P_{AO}$ ) profile controlled by $P_{High}$ , the peak $P_{AO}$ during inhalation, $P_{Low}$ , the pressure during exhalation, and $T_{High}$ , the duration of inhalation.

To explore ARDS, we set $0.2\leq \phi_{surf}\leq 0.5$ , $T_{High}=4\;s$ , and $P_{High}=20\;cmH_{2}O$ to achieve optimal lung compliance during ventilation for these simulated surfactant conditions (Putensen and Tobin, 2013). We note that this value of $P_{High}$ is suitable for the range of lung compliances in this model with this range of $\phi_{surf}$ . We investigate this model’s response to ventilation protocols by manipulating the parameter most likely to influence recruitment/derecruitment (RD):

• ***T***_***Low***_^***+***^: 25% MEFR provides a long $T_{Low}$ , and thus an increased likelihood of derecruitment, and
• ***T***_***Low***_^***−***^: 75% MEFR conversely provides a short $T_{Low}$ , providing little time for derecruitment.

**FIGURE 4:**
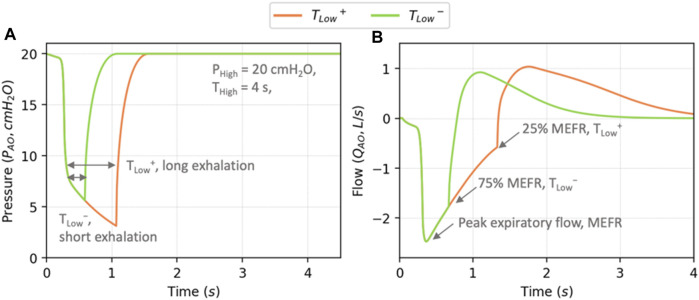
Example profiles of airway opening pressure $P_{AO}$ **(A)** and flow **(B)** using TCAV with $\phi_{surf}=0.5$ . Exhalation is terminated when the magnitude of exhalation exceeds a fraction of the maximum expiratory flow rate (MEFR). *T*_*Low*_^*+*^ (orange) represents flows extending to 25% MEFR, so RD is likely, while *T*_*Low*_^*−*^ (green) ceases expiration quickly (75% MEFR), and thus reduces RD.

$P_{AO}$ and flowrate for these two settings are shown in Figure 4. During exhalation, the magnitude of $Q_{\exp}$ increases rapidly to the MEFR, and then decreases due to an increase in compliance and resistance as the lung volume decreases After the magnitude of $Q_{\exp}$ decreases below the specified fraction of MEFR, exhalation ceases, and inhalation commences.

The ARDS model was initiated with all airways and alveoli in the open state, with the surfactant level $\phi_{surf}$ applied uniformly. Mechanical ventilation was simulated using *T*_*Low*_^*+*^ or *T*_*Low*_^*−*^ protocols, with ventilation occurring for 60 s, at which point the simulations had reached a stationary state. Results shown in the sections below are from the last full breathing cycle of the simulation. A linear $P_{PL}$ gradient of $0.54\;cmH_{2}O∙{cm}^{-1}$ is applied to simulate supine positioning ventilation (Tawhai et al., 1985) and the average $P_{PL}$ is - $2.1\;cmH_{2}O$ .

## 3 Results and discussion

In this section, we show results from the *T*_*Low*_^*+*^ and *T*_*Low*_^*−*^ protocols for different levels of $0.2\leq \phi_{surf}\leq 0.5,$ where $\phi_{surf}$ represents the fraction of surfactant mass compared to a healthy lung. We first demonstrate the global behavior of the system (tidal volume, pressure-volume loops, and surface tension variation), and then we demonstrate the propensity for RD behavior in the injured lung. Finally, we investigate the predicted energy dissipation due to airflow, tissue viscoelasticity and surfactant dynamics, and recruitment.

### 3.1 Lung volumes

**FIGURE 5:**
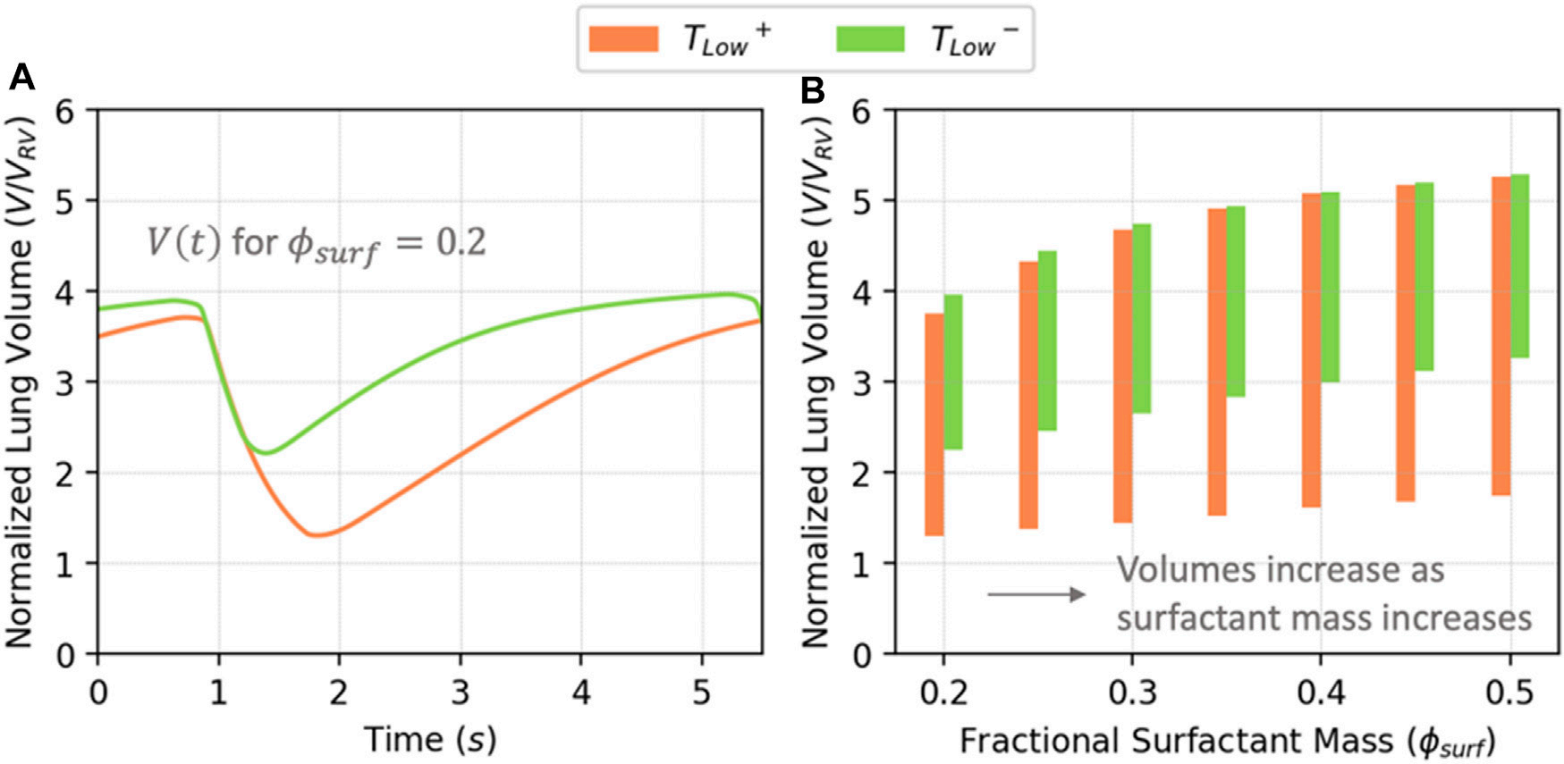
**(A)** Normalized lung volume vs. time for $\phi_{surf}=0.2$ ; **(B)**. Lung volume ranges in the simulated cases as functions of $\phi_{surf}$ . Lung volume increases as $\phi_{surf}$ increases.

Figure 5A shows the behavior of V(t) for *T*_*Low*_^*+*^ and *T*_*Low*_^*−*^ with $\phi_{surf}=0.2$ , while Figure 5B demonstrates how the volume range varies as a function of $\phi_{surf}$ . Overall, with increasing surfactant concentration $\phi_{surf}$ , the maximum and minimum volumes increase, with the tidal volumes asymptotic to a constant level as $\phi_{surf}$ increases.

**FIGURE 6:**
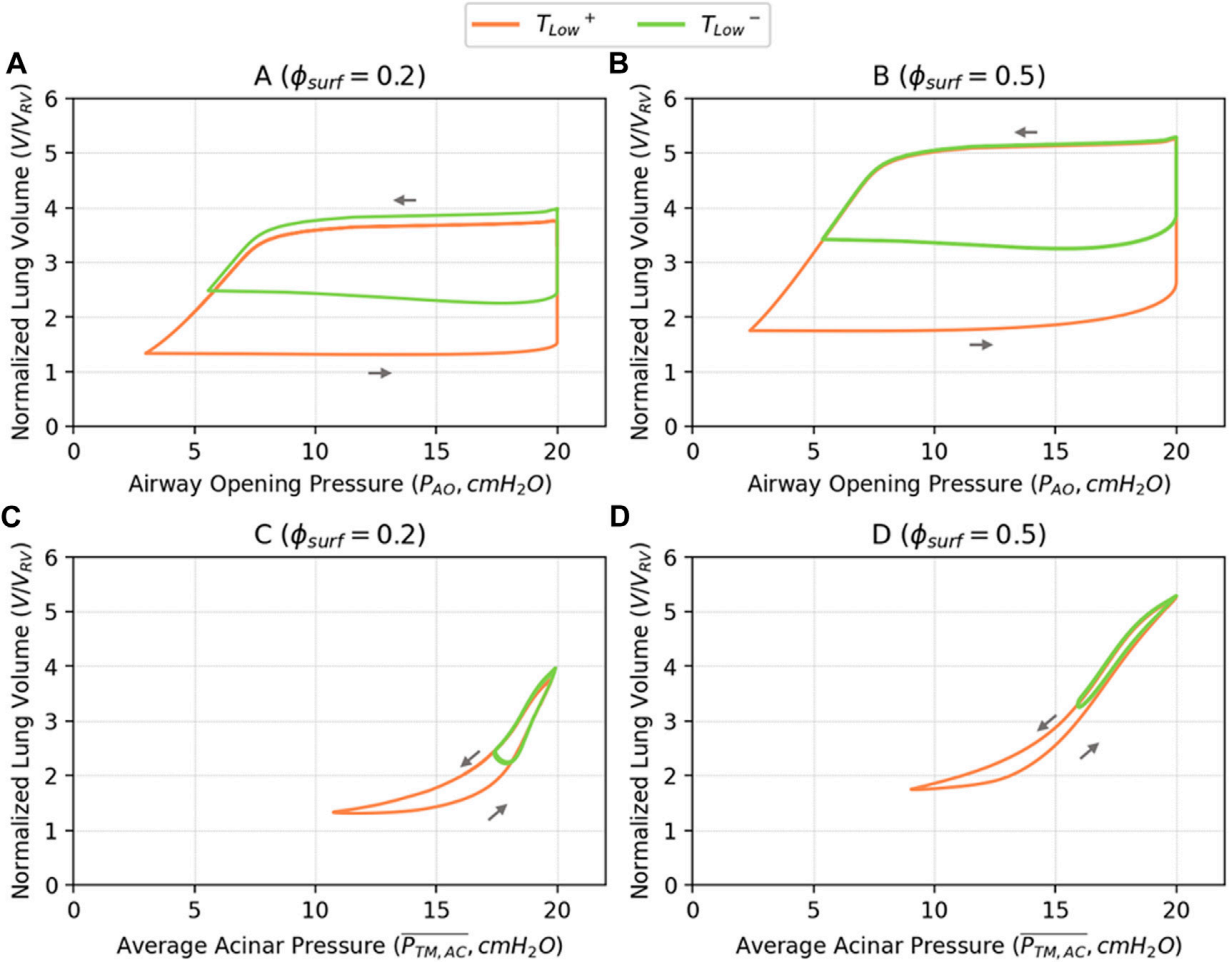
Pressure-volume relationships based on airway opening pressure ( $P_{AO}$ , **A, B**), and average acinar pressure ( $\bar{P_{AC}}$ , **C, D**). The $P_{AO}V$ shown in the **(A, B)** illustrate the global PV behavior, which is affected by both the airway network resistance and local alveolar behavior. The $\bar{P_{AC}}V$ shown in the **(C, D)** illustrate the local PV behavior in the alveolar region. Alveolar compliance shown in the **(C, D)** is greatly increased with $\phi_{surf}=0.5$ compared to $\phi_{surf}=0.2$ .

Figure 6 illustrates PV relationships for $\phi_{surf}$ of 0.2 and 0.5. In Figures 6A,B, the complete $P_{AO}V$ relationship is shown, while in Figures 6C,D the airway component is removed leaving only the alveolar $\bar{P_{AC}}V$ relationship. The full $P_{AO}V$ loops exhibit significantly greater area than the alveolar $\bar{P_{AC}}V$ loops, indicating substantial energy dissipation through airflow. In Figures 6C,D, increasing $\phi_{surf}$ expands the acinar pressure range, decreases the minimum $P_{AC}$ due to reduced surface tension, and increases the slope at high pressure, indicating increased lung compliance.

### 3.2 Surfactant behavior

**FIGURE 7:**
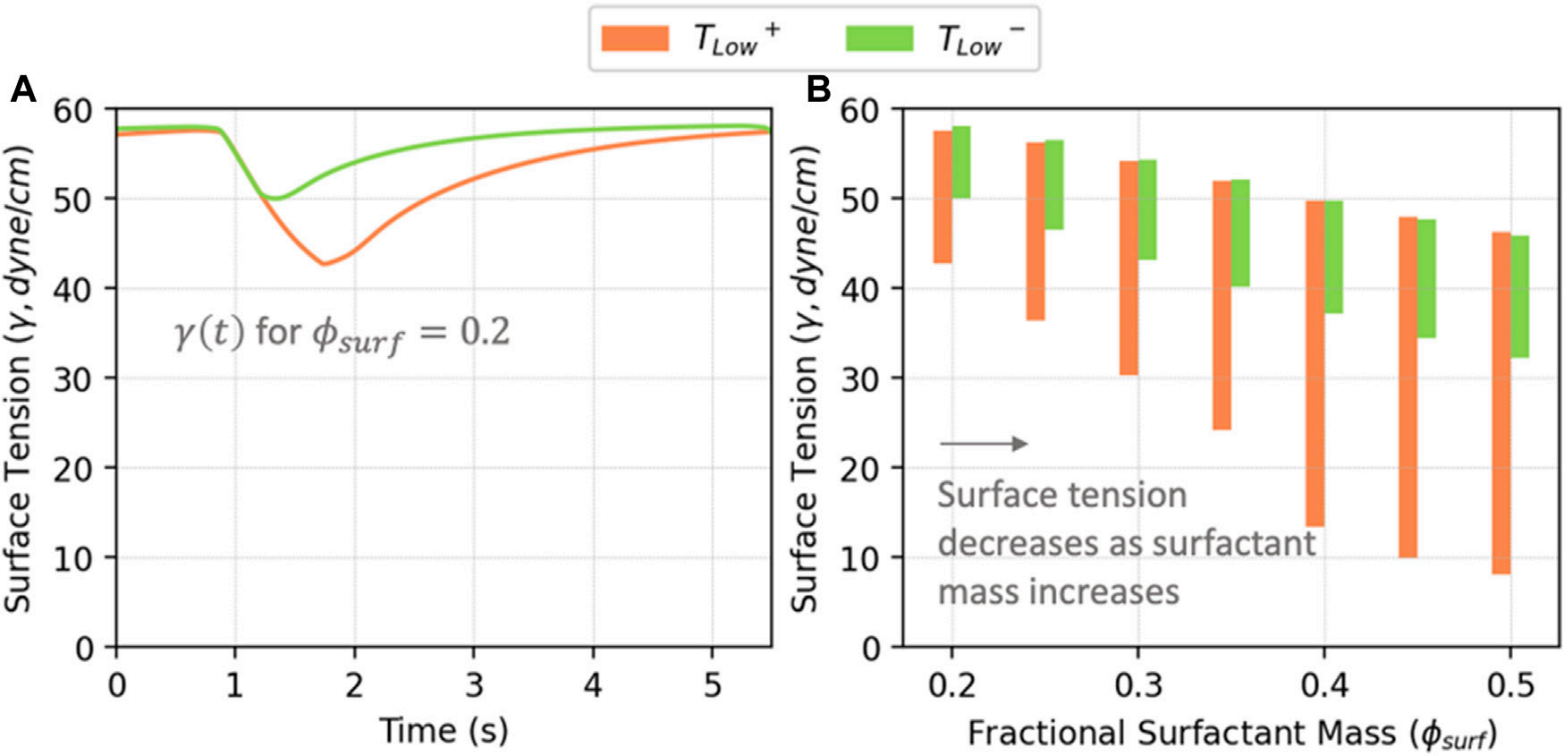
**(A)** Average surface tension vs. time in acini for $\phi_{surf}=0.2$ ; **(B)**. Surface tension from simulated cases. Surface tension decreases as the surfactant conditions improve (higher $\phi_{surf}$ ). The range of surface tension from *T*_*Low*_^*+*^ is greater than *T*_*Low*_^*−*^ due to higher tidal volumes.

Figure 7 illustrates the correlation between $\phi_{surf}$ and alveolar surface tension ( γ ) for *T*_*Low*_^*+*^ and *T*_*Low*_^*−*^ ventilation. Figure 7A displays the time-dependent behavior $\gamma \left(t\right)$ for $\phi_{surf}=0.2$ , while Figure 7B represents the range of values for different $\phi_{surf}$ . Unsurprisingly, smaller $\phi_{surf}$ results in higher surface tension, particularly in the case of *T*_*Low*_^*−*^ ventilation. As $\phi_{surf}$ increases, the overall surface tension decreases, which significantly affects the tidal volume (Figure 5). Moreover, with increasing $\phi_{surf}$ , the surface tension continues to decrease. In the case of long exhalation (*T*_*Low*_^*+*^) ventilation with $\phi_{surf}=0.5$ , $\gamma_{\min}$ nearly approaches multilayer formation values. This reduced surface tension could be protective in so far as it may reduce interfacial instabilities that lead to derecruitment, though in fact it is not sufficient, as will be demonstrated next.

### 3.3 Recruitment and derecruitment

**FIGURE 8:**
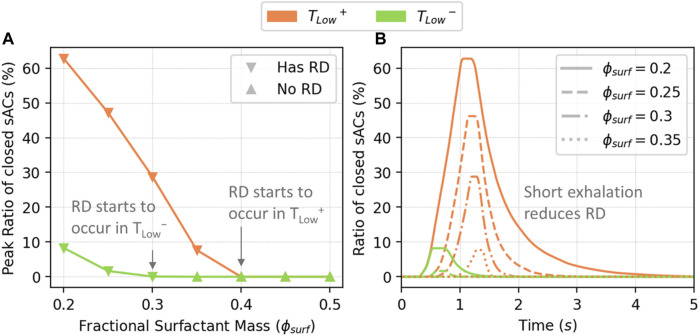
The peak percentage of closed sACs **(A)**, and closed sACs vs. time **(B)**. RD starts to occur in *T*_*Low*_^*+*^ when $\phi_{surf}\leq 0.4$ , and in *T*_*Low*_^*−*^ $\phi_{surf}\leq 0.3$ . The time series shown in the right panel shows that *T*_*Low*_^*−*^ reduces derecruitment through a reduction in exhalation time.

Figure 8A illustrates the recruitment/derecruitment patterns obtained by ventilating with *T*_*Low*_^*+*^ and *T*_*Low*_^*−*^ in relation to $\phi_{surf}$ . In Figure 8A, the peak ratio of closed sACs during ventilation shows that *T*_*Low*_^*−*^ mode is protective, with fewer events than *T*_*Low*_^*+*^. As the amount of surfactant decreases, RD events begin at $\phi_{surf}\leq 0.4$ in *T*_*Low*_^*+*^ and $\phi_{surf}\leq 0.3$ in *T*_*Low*_^*−*^. Figure 8B demonstrates that even with very low surfactant levels ( $\phi_{surf}=0.2$ ), *T*_*Low*_^*−*^ has significantly fewer RD events compared to *T*_*Low*_^*+*^ due to its shorter exhalation time (0.6s in *T*_*Low*_^*−*^ vs 1.4s in *T*_*Low*_^*+*^).

**FIGURE 9:**
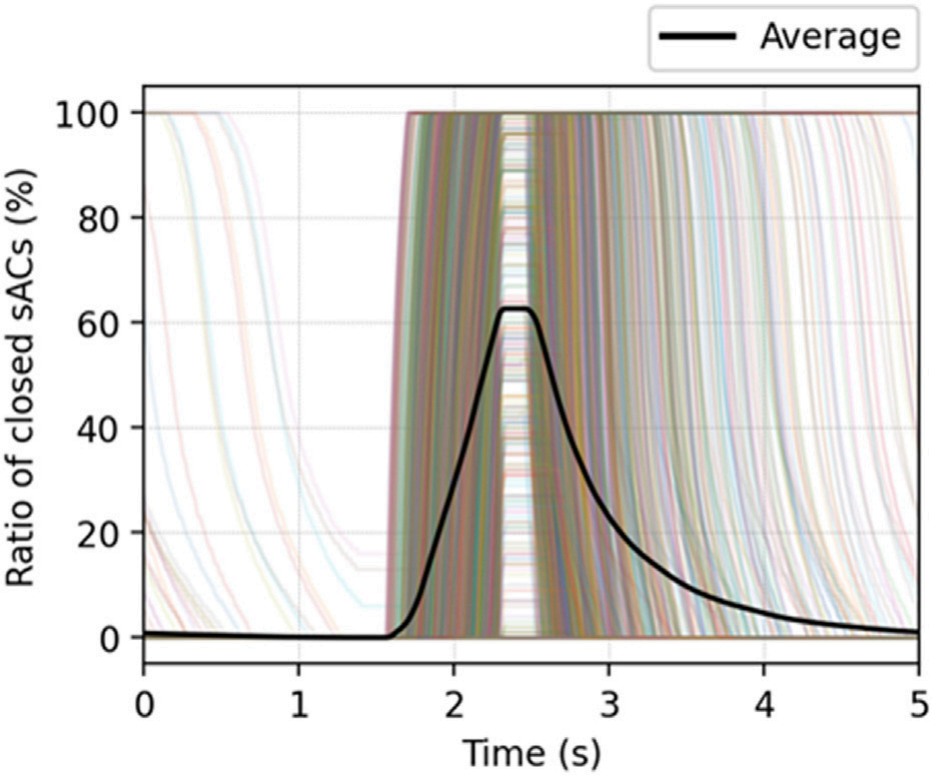
The percentage of closed sACs in the acinar regions from the simulation of $\phi_{\text{surf}}=0.2$ with TLow + ventilation mode. Each opaque colored line represents an acinus, and the solid black line represents the average of the entire system. The RD behavior in local acinar regions is highly heterogeneous, which is a result from the structural differences that exist among airways and acini.

We note that the global behavior shown in Figure 8B is a result from the local acinar behavior that is highly heterogeneous. Figure 9 demonstrates the percentage of closed sACs in the acinar regions from the simulation of $\phi_{surf}=0.2$ with *T*_*Low*_^*+*^ ventilation mode. The data for each acinus is represented by an opaque colored line (n = 1364), while the average behavior of the whole system is represented by the solid black line. This demonstrates how the closure and reopening of sACs occur at different times in each acinus as a result of the structural inhomogeneity that exists among airways and acini, which have been analyzed in our previous model (Ma et al., 2020). Although not explored in this study, such heterogeneity may lead to raised stress/strain concentration and thereby exacerbate lung injury (Nieman et al., 2018; Gaver et al., 2020).

Our model demonstrates dynamics that are representative of an ARDS lung, including elevated pulmonary elastance and recruitment events. Herein we examine the implications and limitations of this model.

### 3.4 Energy dissipation

Recently, there has been a great deal of interest in the hypothesis that energy delivery and/or dissipation during mechanical ventilation is responsible for ventilator-induced lung injury (VILI) (Gattinoni et al., 2017; Barnes et al., 2018; Barnes and Enk, 2019). This is reasonable since energy is required to disrupt and damage any mechanical system. In the context of the lung, energy dissipation could disrupt regions of the lung and spawn further catastrophic damage (Gaver et al., 2020). During mechanical ventilation, the total work of breathing represents the energy dissipated throughout a breathing cycle ( $E_{total}$ ). In this model, we divide the energy dissipation into two partitions associated with regions of the lung:

1) $E_{AW}$ , the energy dissipation due to airflow in the airway network,
2) $E_{AC}$ , the energy dissipation in the acini due to alveolar viscoelasticity, surface tension, and RD.

While these components are difficult to assess *in vivo*, our integrated multiscale model can provide a testbed to evaluate the relative contributions and locations of each.

The total energy dissipation $E_{total}$ and partitions can be evaluated from the macroscale PV loops (Figure 6):

$$E_{total}=E_{AW}+E_{AC}=\oint P_{AO}dV,$$ (14)

$$E_{AC}=\oint \bar{P_{AC}}dV.$$ (15)

**FIGURE 10:**
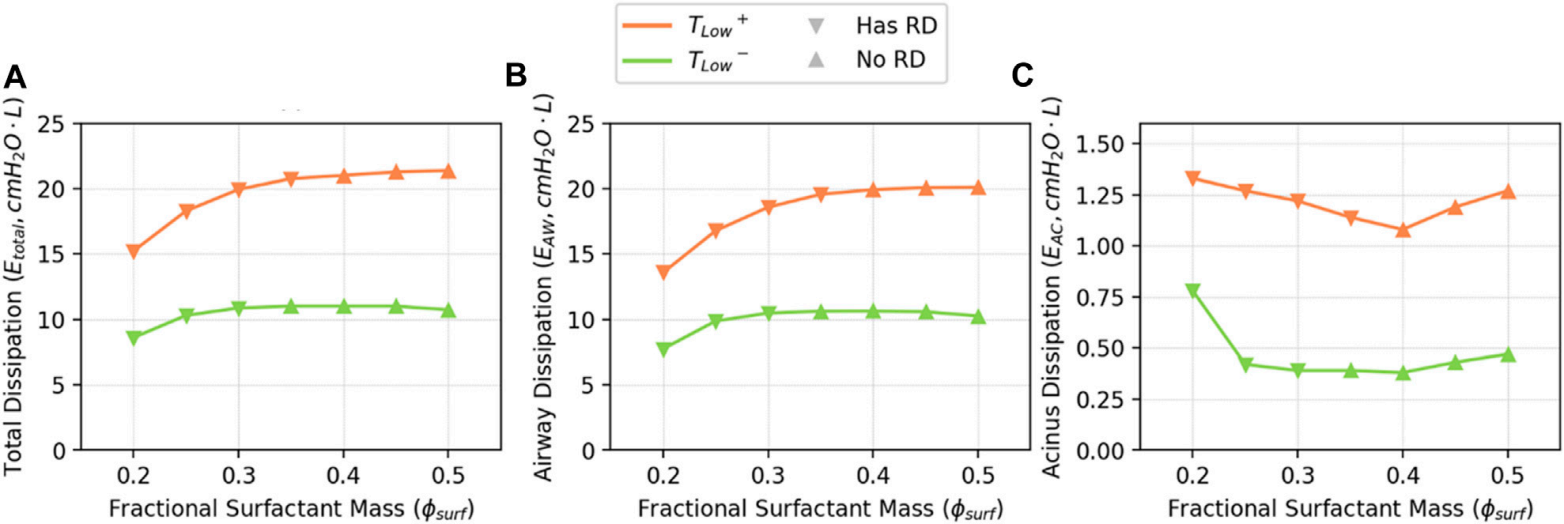
Estimated $E_{total}$ **(A)** and dissipation partitions, which are $E_{AW}$ **(B)**, and $E_{AC}$ **(C)**. $E_{AW}$ is proportional to tidal volume and appears unaffected by RD, as the properties of the upper airway network are not changed by ARDS in this study. $E_{AC}$ is significantly higher when RD exists, which is a result from increased elastance and dissipation due to recruitment ( $E_{RD}$ ).

Figure 10 demonstrates $E_{total}$ and dissipation partitions as a function of $\phi_{surf}$ . Comparing Figures 10A,B, it is evident that the great majority (90%–96%) of energy dissipation occurs due to airflow ( $E_{AW}$ ), and that this correlates with tidal volumes shown in Figure 5B. Figure 10C shows that the remainder of the energy dissipation is due to acinar viscoelastic/surfactant and RD dissipation ( $E_{AC}$ ), and this form of dissipation is much greater for *T*_*Low*_^*+*^ than *T*_*Low*_^*−*^. It is notable that there is an increase in $E_{AC}$ when RD occurs, and this is attributed to the increased elastance of the “baby acinus” (Figure 3) that exists with alveolar derecruitment. This may be related to concentrated mechanical stresses that could be deleterious.

**FIGURE 11:**
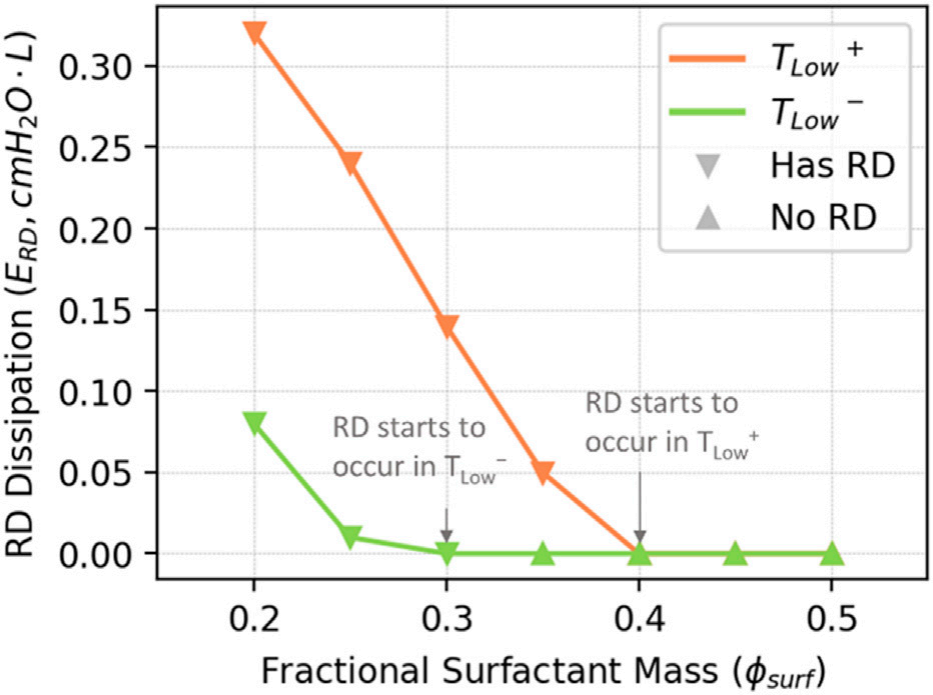
Estimated $E_{RD}$ from Eq. 13. $E_{RD}$ is proportional to the RD level shown in Figure 8A, where RD to occur in *T*_*Low*_^*+*^ when $\phi_{surf}\leq 0.4$ , and in *T*_*Low*_^*−*^ $\phi_{surf}\leq 0.3$ . $E_{RD}$ contributes to the increase of $E_{AC}$ in Figure 10 when RD occur.

In Figure 11, we illustrate the estimated energy dissipation resulting from airway and alveolar recruitment ( $E_{RD}$ ), and note that this accounts for as much as 25% of $E_{AC}$ , but only accounts for ∼2% of $E_{total}$ . RD causes atelectrauma, which has been identified as an important contributor to VILI (Seah et al., 2011; Ramcharran et al., 2022).

Given its small contribution to total energy dissipation, how can $E_{RD}$ be a major contributor to ARDS? Its significance becomes apparent when considering its concentrated nature and brief duration ( ∆t ∼ msec). The power ( $E_{RD}/∆t)$ and power intensity ( $E_{RD}/\left(A*∆t\right))$ associated with $E_{RD}$ are likely to surpass those of $E_{AC}$ , unless there is an extraordinary level of overdistension. Additionally, $E_{RD}$ is expected to have greater relevance than $E_{AC}$ unless airflow-induced shear stresses cause damage. Furthermore, in the ARDS-afflicted lung, where recruitment and derecruitment occur, the pathological stresses on the lung tissue tend to concentrate at the interface between open and closed alveolar regions (Cressoni et al., 2015). By utilizing our model, it becomes possible to quantitatively estimate and predict the extent to which energy dissipation becomes concentrated during mechanical ventilation.

### 3.5 Surfactant dynamics

**FIGURE 12:**
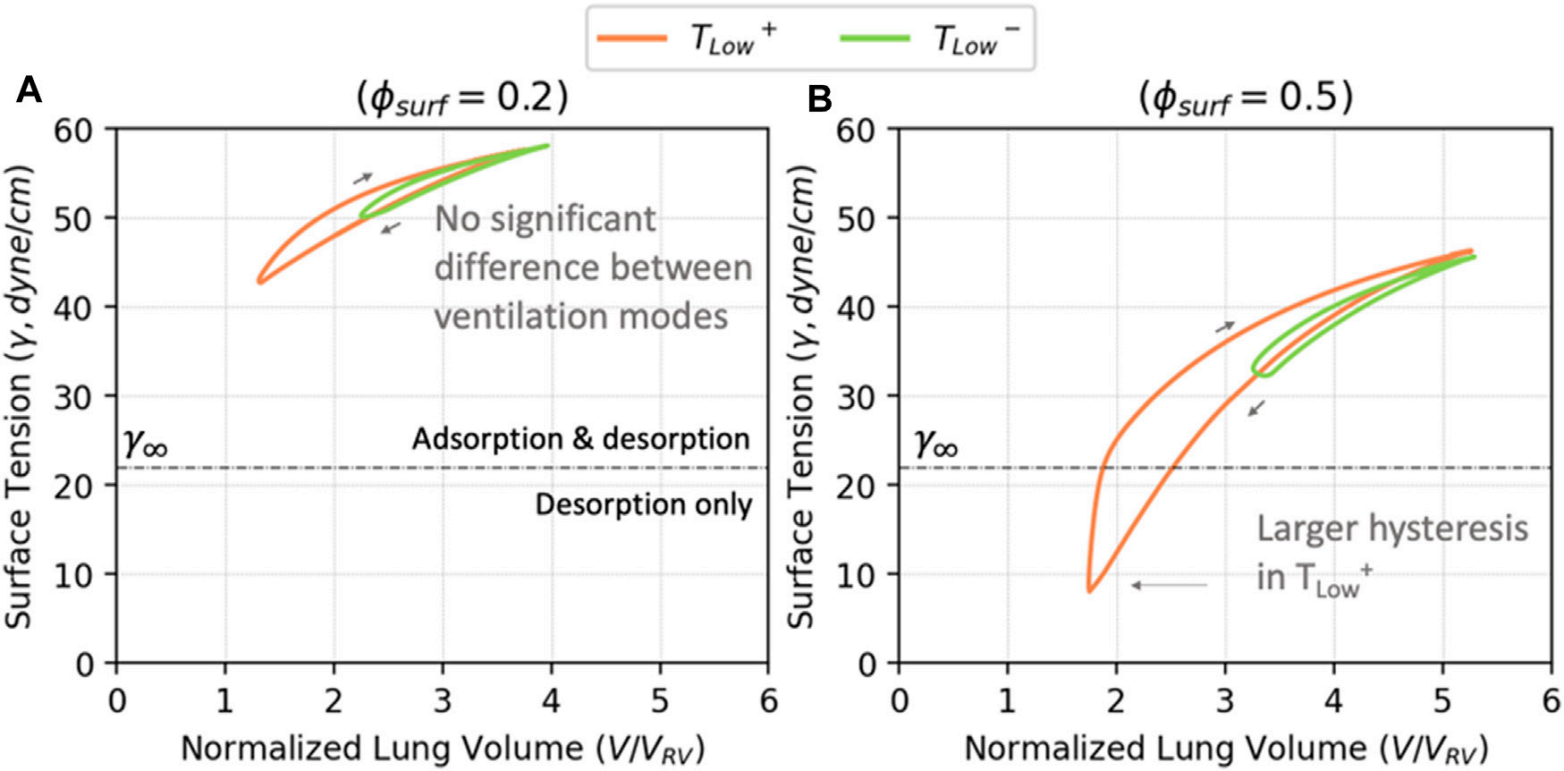
The volume—surface tension relationship for $\phi_{surf}=0.2$ **(A)** and $\phi_{surf}=0.5$ **(B)**. The overall level of surface tension corresponds to Figure 5. For $\phi_{surf}=0.2$ , the amount of surfactant in the system is too low to trigger the change in surfactant dynamics when $\gamma <\gamma_{\infty }$ . The surfactant hysteresis is small, and no significant differences can be found between the two ventilation modes. In $\phi_{surf}=0.5$ , the amount of surfactant in the system is enough for adsorption to stop when $\gamma <\gamma_{\infty }$ in *T*_*Low*_^*+*^. This leads to a larger surface tension hysteresis.

To understand the implications of RD energy dissipation, it is necessary to elucidate how surfactant dysfunction and lung pathophysiology interact. As shown in Figure 7B, as the amount of surfactant in the system increases, both the maximum and minimum surface tensions decrease, as does the surface tension range. We demonstrate this with $\phi_{surf}=0.2$ and $\phi_{surf}=0.5$ as examples yielding the volume-surface tension relationships shown in Figure 12.

Figure 12A demonstrates that for $\phi_{surf}=0.2$ , the surface tension is always greater than $\gamma_{\infty }$ (the equilibrium surface tension). In this regime, a dynamic equilibrium exists between adsorption and desorption. Consequently, the exchange between the surface and bulk phases results in a small degree of surface tension hysteresis. Figure 12B demonstrates what happens with a higher concentration, $\phi_{surf}=0.5$ . In this case, the surface tension becomes sub-equilibrium (lower than $\gamma_{\infty }$ ) in *T*_*Low*_^*+*^, but not in *T*_*Low*_^*−*^. Therefore, even though $\phi_{surf}$ is increased, for *T*_*Low*_^*−*^ the surfactant deviation is constrained because ${\gamma >\gamma }_{\infty }$ , and hence both adsorption and desorption behavior exist (similar to $\phi_{surf}=0.2)$ . In contrast, with *T*_*Low*_^*+*^, the increased amount of surfactant allows $\gamma <\gamma_{\infty }$ at low lung volumes (V/V_RV_ < 2.5). In this case, during the exhalation phase, adsorption ceases and only surfactant desorption occurs. Nevertheless, despite the lack of adsorption, the reduction of the surface area increases the surface concentration and reduces the surface tension, resulting in an increased surface tension hysteresis.

**FIGURE 13:**
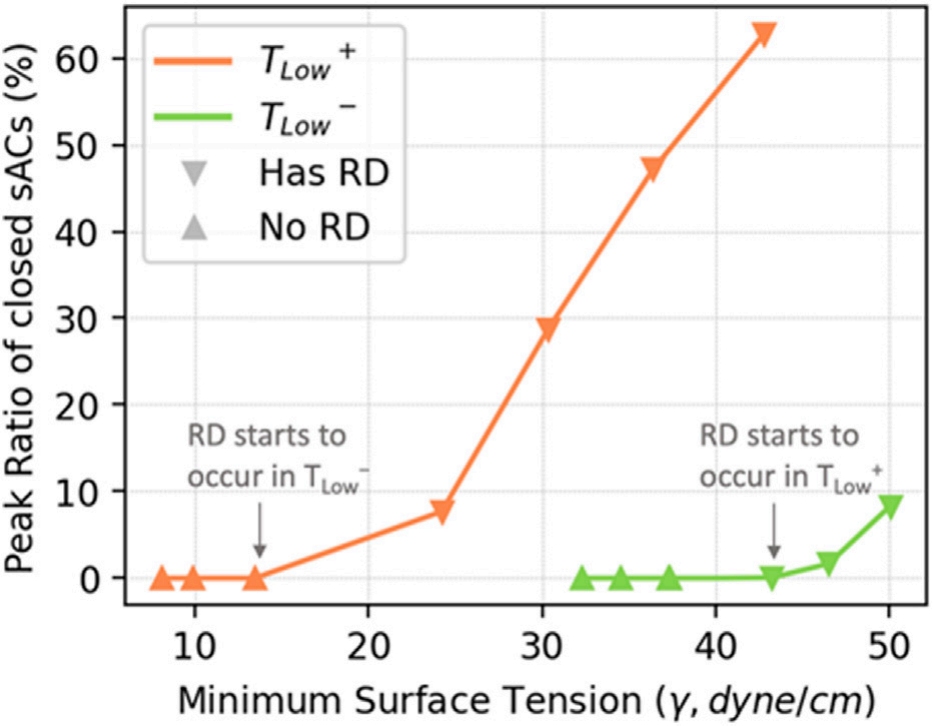
The relationship between minimum surface tension and the peak level of RD in simulated cases. The peak level of RD is proportional to the minimum surface tension within each ventilation mode.

The hysteresis loops in Figure 12 show that *T*_*Low*_^*+*^ results in lower minimum surface tensions than *T*_*Low*_^*−*^, but this does not directly relate to alveolar stability (Figure 8). Figure 13 explores the relationship between minimum surface tension and the peak RD, revealing the critical minimum surface tension ( $\gamma_{crit}$ ) associated with derecruitment. For *T*_*Low*_^*+*^, $\gamma_{crit}$ = 13 dyne/cm, while for *T*_*Low*_^*−*^, $\gamma_{crit}$ = 42 dyne/cm. So, *T*_*Low*_^*−*^ ventilation is more stable even though the surface tensions are higher. This behavior contradicts the notion that low γ alone stabilizes the lung. In the mechanically ventilated unhealthy lung, the correlation between low surface tension and stability is complex and depends on multi-scale interactions involving surfactant quantity ( $\phi_{surf}$ ) and ventilation processes. Increasing $\phi_{surf}$ is clearly protective, but lowering the minimum surface tension through ventilation alone is not. In the case of *T*_*Low*_^*+*^ ventilation, despite lower γ , more RD events occur due to prolonged exhalation, resulting in decreased stability by lowering lung volume and allowing time for derecruitment. This result emphasizes the fact that ventilation timing, not pressures alone, is critically important in protective ventilation modalities (Ramcharran et al., 2022).

### 3.6 Limitations

Our model has limitations that require consideration to assess the validity of the predictions. These limitations stem from the reliance on a substantial number of parameters, some of which are inferred on the basis of known physiological but lack experimental reference values or may vary within the lung or among patients. We estimate the values of these parameters through idealized assumptions that provide physiologically reasonable behavior in a healthy lung model. Our multi-scale model integrates reduced-dimension representations of micro-scale phenomena. Although these models are grounded in physics, they simplify the dynamic physicochemical interactions at the smallest scales, such as recruitment dynamics, despite the large number of interactions.

In developing our ARDS model, we made rational and simplified modifications to our existing healthy lung model. For instance, we addressed surfactant dysfunction by globally reducing surfactant function using the parameter $\phi_{surf}$ . However, this approach does not consider the physicochemical process of regional competitive adsorption (Fernsler and Zasadzinski, 2009), or account for the heterogeneous nature of ARDS.

To model RD, we adapted a time-dependent model (Massa et al., 1985) by incorporating physically justifiable opening and closing pressures, and trajectory velocities. This model used a single acinar entrance region connected to subtended sub-acinar units (sACs) that exhibit fractional measures of RD. However, this representation does not account for potential interactions between acini, the airflow among alveolar regions and liquid fluxes. Another variation of this trajectory model has recently been published in (Geitner et al., 2022).

The current modeling approach does not account for the microscale fluid dynamics occurring in the small airways, the surfactant fluxes, and the interfacial instabilities of the lining liquid. These factors can trigger the development of liquid plugs. Plugs can also cause airway collapse. Recruitment of collapsed airways is achieved by a finger of air that separates the walls that are held in apposition. These types of events can increase flow resistance dramatically. While single events of this nature have been modeled computationally (Halpern and Grotberg, 1992; Gaver et al., 1996; Halpern and Gaver, 2012; Filoche et al., 2015; Fujioka et al., 2016; Ryans et al., 2016), including this level of complexity in a network model is not yet computationally feasible. Consequently, our model cannot currently simulate the microscale fluid mechanical interactions occurring at the time scale of individual recruitment events. As such, the present model does not completely represent the dynamic complexity of the diseased lung, and likely underestimates energy dissipation from RD. In addition, we are neglecting fluid flow across the blood-gas barrier, which can modulate surfactant function. Fluid flow itself can be influenced by surface tension effects that modify Starling pressure gradients. This could influence the course of injury development (Beretta et al., 2021).

Overdistension is represented by viscoelastic models of distensible alveolar tissue stress-strain behavior, however this does not include a damage mechanism that would change the elastic tissue properties and dissipate energy in the process. We also have not yet included mechanical stress concentrations that could exist at the interface between recruited and derecruited alveolar regions, which may lead to microscale tissue distension and tissue damage. This can contribute to ventilator-induced lung injury (VILI) through microfluidic interactions and biomechanical stresses that may lead to injury progression processes such as those proposed by (Gaver et al., 2020).

Nevertheless, this idealized model is employed to investigate and highlight the behavior of ARDS in comparison to a healthy lung and provide insight into biophysical interactions that cannot be experimentally measured. The simulation results demonstrate qualitative similarities to those obtained from animal experiments (Nieman et al., 2020). The future validation and improvement of this model will rely on the availability of relevant real-world data and the efficacy of parameter fitting.

## 4 Conclusion

In this study, we present a computational model to investigate the multiscale network behavior of a lung afflicted with ARDS. To do so, we modified a healthy lung model (Ma et al., 2020), and integrated acinar mechanics that includes viscoelastic tissue behavior and recruitment/derecruitment dynamics. Our model successfully reproduces realistic PV behavior and predicts RD events. Simulations of Time-Controlled Adaptive Ventilation (TCAV) modes with either short or long duration exhalation (*T*_*Low*_^*−*^ and *T*_*Low*_^*+*^, respectively) illustrate a significantly increased incidence of RD for *T*_*Low*_^*+*^ despite the reduction in surface tensions due to interfacial compression. This finding aligns with experimental investigations that emphasize the importance of timing, in addition to pressure, in protective ventilation strategies (Ramcharran et al., 2022).

A benefit of this computational approach is its capacity to evaluate processes that are challenging to measure *in vivo*. We showcase its capacity to evaluate the energy dissipation landscape with a breakdown of energy dissipation from airflow, acinar tissue viscoelastic and surfactant transport, and RD mechanisms. Our simulations reveal that the majority of energy dissipation occurs from airflow, while a mere 2%–3% stems from RD. Nevertheless, the association of RD to atelectrauma in VILI suggests that RD-related energy dissipation may be far more injurious than other forms of dissipation due to its focal nature, resulting in a higher energy transfer intensity.

Our model is able to predict recruitment events that occur in quick succession during inhalation (Figure 8B). This resembles the “avalanche” behavior described in (Suki et al., 1994). However, the microscale pattern of the “avalanches” in (Baudoin et al., 2013; Stewart and Jensen, 2015) requires tracking liquid lining flows and surfactant transport at the airway level, which is not included in our current model. The excessive mechanical stress on epithelial cells due to plug propagation and rupture have been investigated in (Bilek et al., 2003; Kay et al., 2004; Ghadiali and Gaver, 2008) and cannot yet be investigated with our model. Nevertheless, our model provides a multiscale platform that can be expanded by utilizing those studies as “building blocks” by interconnecting the microscale, mesoscale and macroscale interactions to elucidate how physicochemical properties affect this behavior. This may be valuable in designing the ventilation strategy and managing the local recruitment events.

This *in silico* study is based upon parameter estimates that are intended to illustrate the potential of this model paradigm. Despite the non-specific approach, the results show similarity to on-going analyses of animal model experiments of ARDS (Ramcharran et al., 2022). Going forward, it may be feasible to employ this methodology within a physics-inspired machine-learning framework that adheres to fundamental physical conservation principles. The proposed framework has the potential to ascertain parameter values and distributions that enhance the representation of physiological features while also identifying mechanisms through which biomechanical stress concentrations contribute to an increased propensity for lung damage. Consequently, this process may facilitate the development of patient-specific models of mechanically ventilated lungs, which could be leveraged to create personalized ventilation strategies for mitigating VILI.

## Data Availability

The raw data supporting the conclusion of this article will be made available by the authors, without undue reservation.
