# Supplementary material for: Full-lung simulations of mechanically ventilated lungs incorporating recruitment/derecruitment dynamics
Source: Front Netw Physiol. 2023 Nov 2;3:1257710. doi: 10.3389/fnetp.2023.1257710 (PMC10654632; doi:10.3389/fnetp.2023.1257710)
Supplement: Supplementary file 1 [file DataSheet1.docx]

# Glossary

**Global Parameters**

$P_{PL}$: Pleural pressure

$\phi_{surf}$: Surfactant deactivation factor, which is equal to the fraction of the surfactant mass in the system to the amount in a healthy lung

$\mu_{air}$: viscosity of air ($1.86 cmH_{2}O\cdot s$)

**Airway Parameters**

$C_{AW}$: Airflow conductance in an airway

$C_{AW,P}$: Poiseuille airflow conductance in an airway

$G_{eff}$: Parenchymal shear modulus

$L_{AW}$: Length of an airway

$n$: Horsfield generation of an airway

$P_{AW}$: Internal air pressure of an airway

$P_{DN}$: Downstream air pressure of an airway

$P_{TM,AW}$: Transmural pressure of an airway

$P_{UP}$: Upstream air pressure of an airway

$P_{\gamma,AW}$: Laplace pressure drop due to surface tension in an airway

$Q_{AW}$: Rate of airflow in an airway

$R_{AW}$: Airway radius

$R_{AW,max}$: Maximum airway radius

$R_{in,AW}$: Radius of the air pathway in an airway (airway radius minus the thickness of liquid lining)

**Acinar Parameters**

$P_{AC}$: Internal air pressure of an acinus

$P_{TM,AC}$: Transmural pressure of an acinus

$P_{\gamma,AC}$: Laplace pressure drop dur to surface tension in an acinus

$Q_{AC}$: Rate of airflow in an acinus

$R_{in,ALV}$: Radius of the air space in an alveolus (alveolar radius minus the thickness of liquid lining)

$V_{AC}$: Volume of an acinus

$V_{AC,RV}$: Residual volume of an acinus

$V_{liquid,AC}$: Volume of lining liquid of an acinus

**Parenchyma Parameters**

$\bar{P_{TP}}$: Volume-weighted average acinar transpulmonary pressure in the surrounding parenchyma of an airway

**Surface Tension Parameters**

$R_{eff}$: Effective radius of curvature

$\gamma$: Surface tension

$\Delta R$: Fractional change of $R_{eff,AC}$

$\Delta V$: Fractional change of alveolar airspace

**Surfactant Parameters**

$c_{bulk}$: Surfactant concentration in the bulk of lining liquid

$j_{c,r,a,d}$: Surfactant flux due to collapse, respreading, adsorption, and desorption, respectively

$M_{1,2,b}$: Surfactant mass in the primary layer, secondary layer, and bulk liquid, respectively

$\Gamma_{max}$: Surfactant concentration in the primary layer when the layer is fully packed by surfactant ($3.3\times{10}^{-4} mg/{cm}^{2}$)

$\Gamma_{1,2}$: Surfactant concentration in the primary layer and secondary layer, respectively

$\Gamma_{\infty}$: Surfactant concentration in the primary layer at the equilibrium state ($3\times{10}^{-4} mg/{cm}^{2}$)

$\gamma_{min}$: Surface tension in the primary layer when the layer is fully packed by surfactant ($4 dyne/cm$)

$\gamma_{0}$: Surface tension of water ($72 dyne/cm$)

$\gamma_{\infty}$: Surface tension in the primary layer at the equilibrium state ($22 dyne/cm$)

**Sub-acinar Unit Parameters**

$C_{sAC,i}$: Airflow conductance of an entrance duct

$C_{sAC,open}$: Theoretical airflow conductance of an entrance duct when it is fully open

$C_{E}$: Combined airflow conductance of all entrance airways

$P_{E}$: Air pressure of the entrance airways at the terminal airway end

$P_{C,O}$: Critical pressure for a “virtual trajectory” starts to close and reopen, respectively

$P_{TM,E}$: Transmural pressure of the entrance airways at the terminal airway end

$Q_{AW,t}$: Rate of airflow of the terminal airway attached to the acinus

$S_{C,O}$: Speed for a “virtual trajectory” to close and reopen, respectively

$f$: The position of a “virtual trajectory”

$\overline{f}$: Average position of all the “virtual trajectories” in an acinus

**Mechanical Ventilation Parameters**

$P_{AO}$: Airway opening pressure

$Q_{AO}$: Airway opening flow rate

$P_{High}$: Peak airway opening pressure during inhalation

$Q_{exp}$: Rate of expiratory airflow at the trachea

$Q_{exp,peak}$: Peak rate of expiratory airflow at the trachea

$T_{High}$: Duration of the inhalation

T_Low_: Adaptive duration of the exhalation, which is equal to the ratio of $Q_{exp}/Q_{exp,peak}$ when the exhalation is cut off

# Appendix

This appendix provides more complete details of our model.

## Airflow Conductance

To calculate $C_{AW}$, we applied the result from the computational fluid dynamics study performed by [1], which is based on the original research of [2]. In these studies, the air flow through airway tree bifurcations is simulated to estimate the viscous pressure drop. The $C_{AW}$ expressions are

| $C_{AW,P}=\frac{\pi{R_{in,AW}}^{4}}{8\mu_{air}L_{AW}},$ | (1) |
| --- | --- |
| $C_{AW}=\frac{C_{AW,P}}{K_{n}\sqrt{Re\frac{2R_{in,AW}}{L_{AW}}}},$ | (2) |

where $C_{AW,P}$ is the Poiseuille airway air conductance, $R_{in,AW}$ is the radius of the air pathway, $\mu_{air}$ is the viscosity of the air, $L_{AW}$ is the length of the airway, $K_{n}$ is a constant dependent on the airway Horsfield generation ($n$) following [1], $Re$ is the airway air flow Reynolds number. We note that we only applied the values of $K_{n}$ for the Horsfield generations shown in Table 1 to match the tube law parameters used in [3].

| Horsfield Generation ($n$) | $K_{n}$ | $E_{n}$ | $F_{n}$ |
| --- | --- | --- | --- |
| 1-11 | 0.303 | 0.060 | -0.367 |
| 12 | 0.175 | 0.058 | -0.093 |
| 13 | 0.295 | 0.044 | 0.105 |
| 14 | 0.244 | 0.037 | 0.349 |
| 15 | 0.239 | 0.027 | 0.649 |
| 16 | 0.162 | 0.021 | 0.831 |

Table 1. Airway constants dependent on its Horsfield generations ($\boldsymbol{n}$).

## Tissue Compliance

### Airway Compliance

The bronchial tree consists of airways that are assumed to be compliant tubes with uniform radii at each generation. The morphology of the pulmonary airways was based on [4]. The compliance is described by the pressure-radius relationship, *i.e.*, airway tube law, defined as

| $\frac{R_{AW}}{R_{AW,max}}=\sqrt{0.5\times\left[ 1+\mathrm{erf} \left( E_{n}P_{TM,AW}+F_{n} \right) \right]},$ | (3) |
| --- | --- |

where $R_{AW}$ is the airway radius, $\mathrm{erf}$ is the error function, and $E_{n}$ and $F_{n}$ are constants whose values are dependent on the generation of the airway ($n$) as provided in Table 1 [4]. This tube law is a function of the airway transmural pressure defined in Eq.(2) in the main article.

### Alveolar Compliance

The alveoli are connected as clusters (acini) attached to the terminal airways, which surround neighboring airways. Following [5], the PV relationship ($g_{PV}$) of the acinus can expressed as

| $V_{AC,norm}=\frac{V_{AC}}{V_{RV,AC}}=g_{PV}\left( P_{TM,AC} \right)=a+\frac{b}{1+exp\left[ -(P_{TM,AC}-c)/d \right]},$ | (4) |
| --- | --- |

where $V_{AC,norm}$ is the normalized acinar volume, $P_{TM,AC}$ is the acinar transmural pressure described in Eq.(3) in the main article, and $a,b,c,d$ are fitted parameters from finite-element models of alveoli, and we choose $a=1.23,b=4.84,c=13.7,d=2.06$ to match the lung compliance under mechanical ventilation [5]. The total lung capacity equals six-times the RV lung volume, $V_{TLC}\sim6V_{RV}$.

### Alveolar Viscoelasticity

The alveolar compliance describes the PV relationship of acini at the equilibrium state. However, during breathing lung volume changes dynamically and viscoelasticity needs to be modeled. We modeled alveolar viscoelasticity through the three-element Maxwell model (**Figure 1**). The equations describing this model are:

| $P_{TM,AC}=E_{1}V_{AC,norm}+P_{M,AC},$ | (5) |
| --- | --- |
| $\frac{dP_{M,AC}}{dt}=E_{2}\left( \frac{dV_{AC,norm}}{dt}-\frac{P_{M,AC}}{C_{1}} \right),$ | (6) |

where $E_{1}$ is equivalent to the inverse function of $f_{PV}$, $P_{M,AC}$ is the pressure contribution from $E_{2}$ and $C_{1}$, $E_{2}=1.415 cmH_{2}O$, $C_{1}=0.076 cmH_{2}O\cdot s$. These values are determined based on [6]. Since $\frac{dV_{AC}}{dt}$ is equal to the rate of airflow through the acinus $Q_{AC}$, Eqs.(5) & (6) can be rewritten into

| $P_{TM,AC}={g_{PV}}^{-1}\left( V_{AC,norm} \right)+P_{M,AC},$ | (7) |
| --- | --- |
| $\frac{dP_{M,AC}}{dt}=E_{2}\left( \frac{Q_{AC}}{V_{RV,AC}}-\frac{P_{M,AC}}{C_{1}} \right).$ | (8) |

**
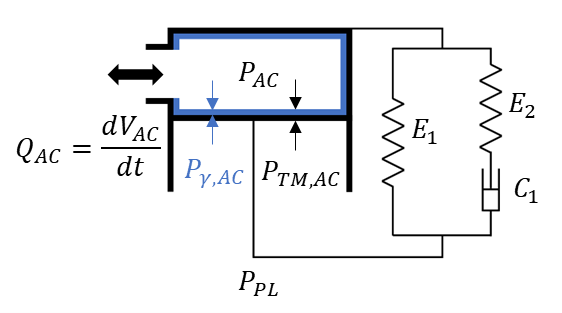
**

**Figure 1.** Viscoelastic model for an acinus, where $E_{1}$ is equivalent to the static PV relationship from [5], and $E_{2}$ and $C_{1}$ are calibrated to [6].

### Parenchymal Tethering

We utilized our previous “pig-in-blanket” airway parenchyma tethering model [7] to simulate the mechanical support to an airway from its surrounding parenchyma. The parenchymal mechanical support is related to its effective shear modulus, $G_{eff}$, given by

| $G_{eff}=Ae^{B\bar{{P_{TP}}^{3}}},$ | (9) |
| --- | --- |

where $\bar{P_{TP}}=\bar{P_{ALV}}-P_{PL}$ is the average transpulmonary pressure in the surrounding acini within the defined region of interest [3, 7], $A=4.75 cmH_{2}O$, $B=5.17\times{10}^{-4}{cmH_{2}O}^{-3}$*.*

## Laplace Pressure Drop

The liquid lining component imposes mechanical stresses on the airways/alveoli due to surface tension through a Laplace pressure drop. In this study, we assume that the alveolar and airway liquid film thickness equals $0.1 \mu m$ at RV [8]. We assume the amount of lining liquid is conserved within acinar and patent airway components and do not model transport of this liquid.

Airways and alveoli films are traditionally treated as cylindrical and spherical shells when modeling pulmonary surface tension, respectively. The Laplace pressure drop due to the surface tension of lining liquid can therefore be expressed as

| $P_{\gamma,AW}=\frac{\gamma}{R_{in,AW}},$ | (10) |
| --- | --- |
| $P_{\gamma,AC}=\frac{2\gamma}{R_{in,ALV}},$ | (11) |

where $R_{in,AW}$ and $R_{in,ALV}$ are the distances from airway center line and alveolar center to the lining film surface, respectively. $R_{in,AW}$ and $R_{in,ALV}$ change following the airway and alveolar size by liquid mass conservation. The surface tension, γ, is a dynamic function of the surfactant concentration described below.

The Laplace pressure drop in the alveolar region determined by Eq.(10) can be rewritten as

| $P_{\gamma,AC}=\frac{\gamma}{R_{eff,AC}},$ | (12) |
| --- | --- |

where $R_{eff,AC}$ is the effective radius of curvature. Following the mass conservation of the lining liquid,

| $\Delta R=\frac{R_{eff,AC}}{R_{ALV,RV}},$ | (13) |
| --- | --- |
| $\Delta V=\frac{V_{AC}-V_{liquid,AC}}{V_{AC,RV}-V_{liquid,AC}},$ | (14) |
| $\Delta R=K_{1}\cdot{\Delta V}^{K_{2}},$ | (15) |

where Eq.(15) describes the relationship between the volume change of an alveolus (represented by the non-dimensional parameter $\Delta V$ in Eq.(13)), and the change of curvature radius (represented by the non-dimensional parameter $\Delta R$ in Eq.(14)) for Laplace’s Law.

If we assume an alveolus is a sphere, and a liquid film coats the interior wall of the sphere uniformly like a “shell”, the Laplace pressure drop determined by Eq.(12) at a radius of $R$ is $P_{drop}=\frac{2\gamma}{R}$. Furthermore, if the volume of the sphere changes by a factor of $\Delta V$, the radius of the sphere will change by a factor of $\Delta R={\Delta V}^{\frac{1}{3}}$. This, in combination with $P_{drop}=\frac{2\gamma}{R}$, is equivalent to $K_{1}=0.5$, and $K_{2}=\frac{1}{3}$.

We find the “spherical shell” assumption” cannot produce physiologically realistic behavior in alveoli. It estimates $P_{\gamma,AC}>6 cmH_{2}O$ at peak tidal breathing lung volume, *i.e.*, FRC (functional residual capacity) + V_t_ (tidal volume), when $P_{AC}-P_{PL}\sim8 cmH_{2}O$ [3]. According to Eq.(4), $P_{TM,AC}$ cannot reach the required pressure level to maintain a lung volume of FRC + V_t._ Therefore, the lung cannot maintain its normal volume oscillation under the spontaneous breathing $P_{PL}$ waveform.

The spherical shell assumption clearly overestimates the alveolar surface tension. Since alveolar surfaces are nearly planar and intersect at a large angle [9] with folding and pleating in the wall [10], the alveolar lining liquid does not cover the interior alveolar surface uniformly. It is therefore not accurate to use the radius of curvature of a spherical shell ($2\gamma/R$) to estimate $P_{\gamma,AC}$ [11]. Therefore, we apply the general form of Eq.(15) and modify the values of $K_{1}$ and $K_{2}$ so that the tidal volume and FRC are reasonable in the simulated healthy lung under spontaneous breathing [12]. We find the combination of $K_{1}=0.59$ and $K_{2}=0.67$ can produce physiologically realistic results.

## Surfactant Multilayer Dynamics

The surfactant multilayer structure includes bulk fluid (with a surfactant mass of $M_{b}$, and a volume concentration of $c_{bulk}$) and two surface layers: a primary layer (with a surfactant mass of $M_{1}$, and a surface concentration of $\Gamma_{1}$); and a secondary layer (with a surfactant mass of $M_{2}$, and a surface concentration of $\Gamma_{2}$).

In this multilayer structure, surfactant adsorption (with a flux term of $j_{a}$) and desorption (with a flux term of $j_{d}$) happen between the bulk liquid (with a surfactant mass of $M_{b}$) and the primary layer (with a surfactant mass of $M_{1}$), and collapse (with a flux term of $j_{c}$) and respreading (with a flux term of $j_{r}$) happen between the primary and secondary layer (with a surfactant mass of $M_{2}$). The governing equations of the surfactant transport are

| $\frac{dM_{1}}{dt}=\left( -j_{c}+j_{r}+j_{a}-j_{d} \right)A,$ | (16) |
| --- | --- |
| $\frac{dM_{2}}{dt}=\left( j_{c}-j_{r} \right)A,$ | (17) |
| $\frac{dM_{b}}{dt}=\left( -j_{a}+j_{d} \right)A,$ | (18) |

where $A$ is the surface area of the air-liquid interface.

The primary layer is in direct contact with the air-liquid interface where surfactant reduces the surface tension, while the secondary layer is created by the collapse of the primary layer and resides between the primary layer and the bulk fluid. The relationship between $\gamma$ and $\Gamma_{1}$ is

| $\gamma=\left\{ \begin{matrix} \gamma_{0}-m\Gamma_{1}, & if \Gamma_{1}<\Gamma_{\infty} \\ m^{'}\left( \Gamma_{1}-\Gamma_{max} \right)+\gamma_{min}, & if \Gamma_{1}\geq\Gamma_{\infty} \end{matrix} \right.,$ | (19) |
| --- | --- |

where $\gamma_{0}=72 dyne/cm$ is the surface tension of water, $m=1.7\times{10}^{5} dyne\cdot cm\cdot{mg}^{-1}$, $m^{'}=-5.3\times{10}^{5} dyne\cdot cm\cdot{mg}^{-1}$, $\Gamma_{max}=3.3\times{10}^{-4} mg/{cm}^{2}$ is the maximum surfactant concentration, $\gamma_{min}=4 dyne/cm$ is the surface tension when $\Gamma_{1}=\Gamma_{max}$, $\Gamma_{\infty}=3\times{10}^{-4} mg/{cm}^{2}$ is the surfactant concentration at equilibrium state. The surface tension at equilibrium state $\gamma_{\infty}=22 dyne/cm$.

The terms for collapse and respreading are defined as

| $j_{c}=\left\{ \begin{matrix} -\frac{\Gamma_{1}}{A}\cdot\frac{dA}{dt} & if \Gamma_{1}>\Gamma_{\max} \\ 0 & otherwise \end{matrix}, \right.$ | (20) |
| --- | --- |
| $j_{r}=\left\{ \begin{matrix} \frac{\Gamma_{2}}{A}\cdot\frac{dA}{dt} & if \Gamma_{1}<\Gamma_{\infty} \\ 0 & otherwise \end{matrix} \right.,$ | (21) |

where $\frac{dA}{dt}$ is the normalized rate of change of the air-liquid interface area.

The terms for adsorption and desorption are defined as

| $j_{a}=\left\{ \begin{matrix} K_{a}c_{bulk}\left( \Gamma_{\infty}-\Gamma_{1} \right)\left( 1-\frac{\Gamma_{2}}{2\Gamma_{\infty}} \right), & when \Gamma_{1}<\Gamma_{\infty} \\ 0, & otherwise \end{matrix} \right.,$ | (22) |
| --- | --- |
| $j_{d}=K_{d}\Gamma_{1}\left( 1-\frac{\Gamma_{2}}{2\Gamma_{\infty}} \right),$ | (23) |

where $K_{a}=1.7 {cm}^{3}\cdot{mg}^{-1}\cdot s^{-1}$ is the adsorption parameter, $K_{d}=1.7\times{10}^{-2} s^{-1}$ is the desorption parameter, and $\left( 1-\frac{\Gamma_{2}}{2\Gamma_{\infty}} \right)A$ is the effective area of the air-liquid interface [12].

# References

1. van Ertbruggen, C., C. Hirsch, and M. Paiva, *Anatomically based three-dimensional model of airways to simulate flow and particle transport using computational fluid dynamics.* J Appl Physiol (1985), 2005. **98**(3): p. 970-80.

2. Pedley, T., R. Schroter, and M. Sudlow, *The prediction of pressure drop and variation of resistance within the human bronchial airways.* Respiration physiology, 1970. **9**(3): p. 387-405.

3. Ma, H., H. Fujioka, D. Halpern, and D.P. Gaver, *Surfactant-Mediated Airway and Acinar Interactions in a Multi-Scale Model of a Healthy Lung.* Frontiers in Physiology, 2020. **11**(941).

4. Lambert, R.K., *Sensitivity and specificity of the computational model for maximal expiratory flow.* J Appl Physiol Respir Environ Exerc Physiol, 1984. **57**(4): p. 958-70.

5. Fujioka, H., D. Halpern, and D.P. Gaver, 3rd, *A model of surfactant-induced surface tension effects on the parenchymal tethering of pulmonary airways.* J Biomech, 2013. **46**(2): p. 319-28.

6. Ismail, M., A. Comerford, and W.A. Wall, *Coupled and reduced dimensional modeling of respiratory mechanics during spontaneous breathing.* Int J Numer Method Biomed Eng, 2013. **29**(11): p. 1285-305.

7. Ryans, J.M., H. Fujioka, and D.P. Gaver, 3rd, *Microscale to mesoscale analysis of parenchymal tethering: the effect of heterogeneous alveolar pressures on the pulmonary mechanics of compliant airways.* J Appl Physiol (1985), 2019. **126**(5): p. 1204-1213.

8. Bastacky, J., C.Y. Lee, J. Goerke, H. Koushafar, D. Yager, L. Kenaga, T.P. Speed, Y. Chen, and J.A. Clements, *Alveolar lining layer is thin and continuous: low-temperature scanning electron microscopy of rat lung.* J Appl Physiol (1985), 1995. **79**(5): p. 1615-28.

9. Wilson, T.A. and H. Bachofen, *A model for mechanical structure of the alveolar duct.* J Appl Physiol Respir Environ Exerc Physiol, 1982. **52**(4): p. 1064-70.

10. Knudsen, L. and M. Ochs, *The micromechanics of lung alveoli: structure and function of surfactant and tissue components.* Histochem Cell Biol, 2018. **150**(6): p. 661-676.

11. Prange, H.D., *Laplace's law and the alveolus: a misconception of anatomy and a misapplication of physics.* Adv Physiol Educ, 2003. **27**(1-4): p. 34-40.

12. Krueger, M.A. and D.P. Gaver, *A theoretical model of pulmonary surfactant multilayer collapse under oscillating area conditions.* Journal of Colloid and Interface Science, 2000. **229**(2): p. 353-364.
